# Avian tuberculosis identified as the potential disease in an outbreak in wild migratory birds in China

**DOI:** 10.1002/mlf2.12164

**Published:** 2025-02-27

**Authors:** Chunge Zhang, Liang Wang, Cheng Zhang, Ning Zhang, Heting Sun, Dong Chu, Siyuan Qin, Zhenghai Ma, Marina Gulyaeva, Alexander Shestopalov, Wenjun Liu, George F. Gao, Yuhai Bi

**Affiliations:** ^1^ CAS Key Laboratory of Pathogen Microbiology and Immunology, Institute of Microbiology, Core‐facility for Biosafety and Laboratory Animal, Center for Influenza Research and Early‐warning (CASCIRE), CAS‐TWAS Center of Excellence for Emerging Infectious Diseases (CEEID) Chinese Academy of Sciences Beijing China; ^2^ University of Chinese Academy of Sciences Beijing China; ^3^ College of Life Science and Technology Xinjiang University Urumchi China; ^4^ General Station for Surveillance of Wildlife‐Borne Infectious Diseases State Forestry and Grassland Administration Shenyang China; ^5^ Federal Research Center of Fundamental and Translational Medicine, Federal State Budget Scientific Institution, Siberian Branch of Russian Academy of Sciences Novosibirsk State University Novosibirsk Russia; ^6^ The D. H. Chen School of Universal Health Zhejiang University Hangzhou China

## Abstract

This study identifies avian tuberculosis as a potential cause of mass mortality in wild migratory birds in Inner Mongolia, China. Combining meta‐transcriptomic sequencing and histopathological analysis, it reveals one of the rare instances of tuberculosis‐associated outbreaks in avian populations. These findings underscore the importance of surveillance on wildlife diseases to mitigate the risk of interspecies transmission of the disease associated pathogens and their broader implications for biodiversity and public health.

Tuberculosis (TB) is a class of diseases caused by pathogens from the genus *Mycobacterium* and is also the leading cause of human deaths due to infectious diseases globally^1^. In 2022, tuberculosis diseases caused 1.4 million human deaths worldwide^2^. Typical symptoms of tuberculosis include a bad cough lasting 3 weeks or longer, chest pain, and coughing up blood or sputum (phlegm from deep inside the lungs)^3^. The host range of pathogens in *Mycobacterium* is widely distributed from terrestrial mammals^4^ to birds^5^, ^6^. Previous studies have documented infections caused by various species of *Mycobacterium* in wild bird populations. For instance, *Mycobacterium avium* has been frequently reported as a cause of avian tuberculosis in wild birds, leading to significant health impacts such as weight loss, respiratory issues, and eventual death, particularly in immunocompromised or stressed birds^7^, ^8^, ^9^, ^10^, ^11^. However, these reports are often limited to sporadic cases or small outbreaks. Large‐scale mortality events linked to *Mycobacterium* infections in wild bird populations are rarely documented. In addition, tuberculosis‐inducing pathogens can cross the host barrier and be transmitted between humans and animals^12^, posing a potential threat to public health. This study aims to elucidate the etiological agent behind an unprecedented event of mass mortality among migratory birds in Inner Mongolia, China, potentially broadening our understanding of *Mycobacterium*'s host range and environmental resilience.

On August 15, 2018, wild bird deaths began in Dalinor Lake, Inner Mongolia, China. By September 19, 2861 wild birds representing 22 different species had died, with an average of 80 deaths per day. Clinical symptoms of diseased wild birds include physical weakness, lying prone, and tiredness of movement. Moreover, some wild birds discharged blue‐green and loose feces. In the present case, three bird species with the highest number of deaths were the common shelduck (CS, *Tadorna tadorna*), ruddy shelduck (RS, *Tadorna ferruginea*), and black‐headed gull (BHG, *Larus ridibundus*). However, the cause of death was unclear.

Organ specimens were collected from six dead wild birds. After mixing samples from the same organ, 12 samples (lung, intestine, heart, spleen, kidney, liver, and six throat and cloacal swabs from six dead birds) were labelled as Lung, Intestine, Heart, Spleen, Kidney, Liver, CS1‐GK, CS2‐GK, RS1‐K, RS2‐GK, RS3‐GK, and BHG‐1GK (K, throat swab; GK, throat and cloacal mixed swab); the samples were used for meta‐transcriptomic sequencing to determine the cause of death. To ensure the accuracy of our findings, Fastp^13^ was employed for quality control and adapter trimming of the raw sequencing data. Utilizing Fastp, we applied default parameters to remove low‐quality reads and adapter sequences, focusing on maintaining reads with a quality score above Q20. Subsequently, MetaPhlAn2^14^ (version 2.2) was used to quantify the microbiome components in these samples. Sequences from viruses contained in each sample were limited, and no virus was shared by all samples; therefore, the studies focused only on detecting bacteria within the samples. Four species belonging to *Mycobacterium* were detected in each sample. Both *Mycobacterium canettii* and *Mycobacterium orygis* were detected in all 12 samples, and *Mycobacterium tuberculosis* was detected in all samples, except for the intestine, CS1‐GK, and RS2‐GK. *Mycobacterium mungi* was detected in only one sample from RS3‐GK. Some other bacteria were also identified among different samples (Figure 1A). As only RNA was used to perform next‐generation sequencing (NGS) and because of the short length of reads generated from NGS, it was difficult to precisely distinguish the taxonomy at the species level. However, the conclusion that wild birds died of *Mycobacterium* infection was strongly supported by the above evidence.

**Figure 1:**
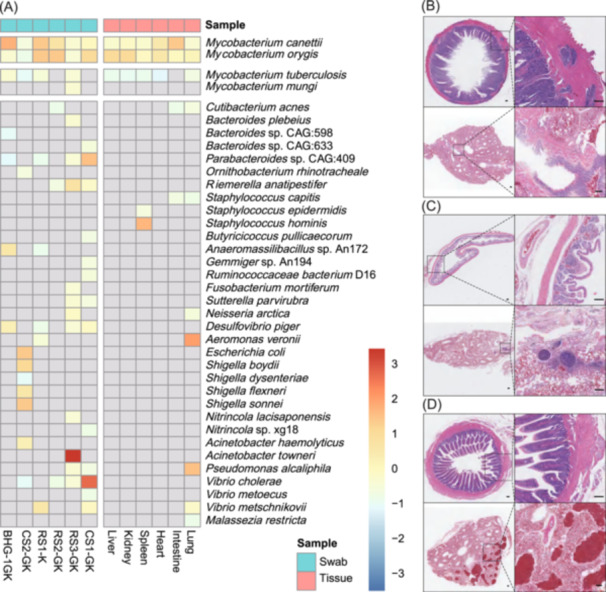
Microbial relative abundance and histopathological changes in affected birds. (A) The heatmap of microbial relative abundance in each sample. Organ samples were collected from six dead wild birds, with the same organs mixed together for meta‐transcriptomic sequencing, as well as the throat and cloacal swabs. Sequencing data from the 12 samples (lung, intestine, heart, spleen, kidney, liver, and six swabs) were analyzed. BHG, black headed gull; CS, common shelduck; K, throat swab; GK, throat and cloacal mixed swab; RS, ruddy shelduck. (B–D) Histopathological changes detected by hematoxylin and eosin (H&E) staining of dead birds. The small intestine and lung tissues of CS (B) and RS (C,D) showed typical lesions, including lymphocyte infiltration, cellulose exudation, intestinal mucosa shedding, and severe congestion of lung tissue. Scale bars, 100 μm.

To further confirm whether the dead birds exhibited typical lesions caused by tuberculosis, we performed histopathological analysis of the tissues. As shown in Figure 1, typical lesions caused by tuberculosis were found in the tissues of CS, including lymphocyte infiltration of the small intestine, cellulose envelope of nodules, and serious congestion of the lungs (Figure 1B). The RS also showed typical lesions such as lymphocyte infiltration, cellulose exudation, intestinal mucosa shedding, and severe congestion of the lung tissue and trachea (Figure 1C). Additionally, we observed a significant influx of inflammatory cells surrounding the bronchioles, accompanied by cellular necrosis and disintegration at the focal center (Figure 1C,D). The histopathological results are consistent with those of the meta‐transcriptomic study. Based on the evidence, we conclude that wild birds in Dalinor Lake, Inner Mongolia, China, likely died of *Mycobacterium* bacterial infection. However, the pathogen of *Mycobacterium* was not successfully isolated so that the animal regression experiment was not performed, which warrants further study.

This study highlights the need for heightened surveillance of *Mycobacterium* in wildlife, particularly in migratory birds, to prevent interspecies transmission and potential public health risks.

## AUTHOR CONTRIBUTIONS

**Chunge Zhang**: Data curation (lead); formal analysis (lead); investigation (lead); methodology (lead); validation (lead); visualization (lead); writing—original draft (lead); writing—review and editing (lead). **Liang Wang**: Data curation (lead); formal analysis (lead); visualization (lead); writing—review and editing (lead). **Cheng Zhang**: Formal analysis (supporting); investigation (supporting). **Ning Zhang**: Methodology (supporting); writing—review and editing (supporting). **Heting Sun**: Data curation (supporting); resource (supporting). **Dong Chu**: Resources (supporting). **Siyuan Qin**: Methodology (supporting); resources (supporting). **Zhenghai Ma**: Resources (supporting). **Marina Gulyaeva**: Methodology (supporting); writing—review and editing (supporting). **Alexander Shestopalov**: Methodology (supporting); writing—review and editing (supporting). **Wenjun Liu**: Resources (supporting). **George F. Gao**: Resources (equal); supervision (equal); writing—review and editing (supporting). **Yuhai Bi**: Conceptualization (lead); funding acquisition (lead); investigation (lead); project administration (lead); resources (lead); supervision (lead); writing—review and editing (lead).

## ETHICS STATEMENT

This study was approved by the Animal Ethics Committee of the Institute of Microbiology, Chinese Academy of Sciences (Approval Number: APIMCAS2018034 and APIMCAS2021112).

## CONFLICT OF INTERESTS

The authors declare no conflict of interests.

## Data Availability

The raw data for this project has been deposited into NMDC (https://nmdc.cn; accession Nos. NMDC40066594‐NMDC40066605) and NCBI (https://www.ncbi.nlm.nih.gov; accession Nos. SRR31509664‐SRR31509675).
